# Antimicrobial Isoflavones and Derivatives from Erythrina (Fabaceae): Structure Activity Perspective (Sar & Qsar) on Experimental and Mined Values Against *Staphylococcus aureus*

**DOI:** 10.3390/antibiotics9050223

**Published:** 2020-04-30

**Authors:** Nicholas J. Sadgrove, Tiago B. Oliveira, Gugulethu P. Khumalo, Sandy F. van Vuuren, Ben-Erik van Wyk

**Affiliations:** 1 Jodrell Science Laboratory, Royal Botanic Gardens, Kew, Richmond, Surrey TW9 3AB, UK; 2 Department of Botany and Plant Biotechnology, University of Johannesburg, P.O. Box 524, Auckland Park 2006, South Africa; khumalogugulethu59@gmail.com (G.P.K.); bevanwyk@uj.ac.za (B.-E.v.W.); 3 Department of Pharmacy, Federal University of Sergipe (UFS-SE), São Cristóvão 491000-000, Brazil; tiago.branquinho@ufs.br; 4 Department of Pharmacy and Pharmacology, Faculty of Health Sciences, University of the Witwatersrand, 7 York Road, Parktown 2193, South Africa; sandy.vanvuuren@wits.ac.za

**Keywords:** pterocarpan, prenylated isoflavonoid, traditional medicine, QSAR, MRSA

## Abstract

Prenylated (iso)flavonoids, -flavans and pterocarpans from taxa in *Erythrina* are repeatedly flagged as potent antimicrobial compounds. In the current study, bark from *E. lysistemon* was extracted and seven isoflavone derivatives were purified: erybraedin A (**1**), phaseollidin (**2**), abyssinone V-4′ methyl ether (**3**), eryzerin C (**4**), alpumisoflavone (**5**), cristacarpin (**6**) and lysisteisoflavone (**7**). Minimum inhibition concentration (MIC) values were determined against a range of species of bacteria (skin pathogens), then values for another 67 derivatives from *Erythrina*, only against *Staphylococcus aureus*, were mined from the literature. Of the seven isolates, MIC values widely ranged from 1–600 μg/mL, with no obvious pattern of selectivity for Gram-types. Nevertheless, using the mined and experimentally determined values against *S. aureus*, Klekota-Roth fragments (Structure Activity Relationship: SAR) were determined then used as molecular descriptors to make a ‘decision tree’ based on structural characters inspired by the classes of antimicrobial potency (classes A-D). Furthermore, to make quantitative predictions of MIC values (Quantitative SAR: QSAR) ‘pace regression’ was utilized and validated (R² = 0.778, Q² = 0.727 and P² = 0.555). Evidently, the position and degree of prenylation is important; however, the presence of hydroxyl groups at positions 5 and 7 in ring A and 4′ in ring B is associated with lower MIC values. While antimicrobial results continue to validate the traditional use of *E. lysistemon* extracts (or *Erythrina* generally) in therapeutic applications consistent with anti-infection, it is surprising that this class of compound is not being utilized more often in general industry applications, such as food or cosmetic preservation, or in topical antimicrobial creams. Prenylated (iso)flavonoids are derived from several other Genera, such as *Dorstenia* (Moraceae), *Ficus* (Moraceae), *Glycyrrhiza* (Fabaceae), *Paulownia* (Lamiales) or *Pomifera* (Moraceae).

## 1. Introduction

*Erythrina* is a common feature of the *materia medica* of many of the world’s cultures, particularly across Africa, the America’s, India, south-east Asia, Australia and the Polynesian Islands. Of the ca. 120 known species, 38 occur in Africa and Madagascar, 70 in the neotropics (mostly South America) and 12 in Asia and Oceania [1]. Salient examples of medicinally important species include *E. variegata* L. in the Indian folkloric tradition [2], *E. mildbraedii* Harms. in Nigerian practice [3], *E. subumbrans* Merr. in south-east Asian practice [4,5] and *E. lysistemon* Hutch. of southern Africa [6], just to name a few.

A vernacular name commonly associated with the species of *Erythrina* is ‘coral’, such as ‘coral tree’ or ‘Indian coral tree’ (*E. variegata*). The etymology of such a name is clearly related to the appearance of the mostly bright red flowers, which can cover leafless branches in the spring and confer the appearance of coral or barnacles.

In the medicinal tradition, *Erythrina* is often included in treatments consistent with infectious diseases. For example, the South African traditional uses of *E. lysistemon* are mostly for treatment of topical sores, abscesses and wounds [6], suggesting both antimicrobial and anti-inflammatory effects [7]. Furthermore, it has been used to treat dysentery and the symptoms of menopause [8], suggesting estrogenic activity [9]. However, in vitro bioassays have gone beyond implications from traditional uses. Pharmacological activities including anti-diabetic activity [10], cholesterol gall-stone prevention [8] and anticancer activities [11,12] have also been demonstrated from the crude extracts of *E. lysistemon*.

Chemical studies of *Erythrina* have assigned a diverse array of biologically active compounds, but the chemistry of the genus is dominated by two classes, one being the erythrinan alkaloids [13] and the other (iso)flavonoid derivatives [3,14,15,16,17,18]. The (iso)flavonoid derivatives are commonly prenylated pterocarpans or prenylated isoflavones, prenylated flavones and flavans. Studies specifically of *E. lysistemon* have demonstrated an abundance of both classes of compound, but prenylated pterocarpans have received the greater focus with regards to the biological activities ascribed in medicinal practice [8,9,10] and erythrinan alkaloids have been previously linked to anticancer activities [12].

Thus, it is the (iso)flavonoid derivatives that are regarded as most important in the antimicrobial outcomes for the therapeutic use of *Erythrina* bark. Generally, antimicrobial testing of prenylated pterocarpans and (iso)flavonoids shows that these classes of compounds are probably among the most potent plant-derived antimicrobial compounds [19]. During the course of studies on antimicrobial compounds from bark of *E. lysistemon*, it became clear that a quantitative structure activity relationship (QSAR) perspective could be derived from analysis of the collective data from the literature and in our research group. Many of these studies adhere to the protocol specified by the Clinical Laboratory Standards Institute (CLSI) [20], meaning they are comparable. 

Due to the lack of absolute specificity in the mechanisms of this class [21], it is clear that variation of minimum inhibition concentration (MIC) values across strains of *S. aureus* are minimal by comparison with standard technical error across studies. For example, strains that derive from emergent resistant mechanisms, such as methicillin resistance *Staphylococcus aureus* (MRSA), demonstrate resistance to specific antibiotics, such as β-lactams, but maintain susceptibility to antimicrobial compounds with generalized activity, or low degrees of specificity, such as those reported for the isoflavones and their derivatives [21]. Generally, membrane disruption is the dominant mechanism of these compounds [22].

Due to a limited number of (iso)flavonoid derivatives available in any one laboratory, a comprehensive QSAR perspective has never been completed against *S. aureus*. However, a study that focused on *Escherichia coli* and *Listeria monocytogenes* managed to make a good QSAR model using a library of 30 different compounds [23], which produced an averaged linear regression with R^2^_m_ between 0.70 and 0.75. In that study, higher polar surface area, globularity and higher molecular flexibility were regarded as significant factors in antibacterial outcomes against both Gram-types, but with a higher number of hydrogen bond acceptors in the Gram-negative *E. coli*. They also validated earlier structure activities relationship (SAR) studies that identified that the position of prenylation on the ring is also a significant factor, where C8 is better than C6 for flavanones and C6 better than C8 for isoflavones [24,25]. Diprenylation is superior to prenylation [22] and the position of two OH substituents ortho to the prenyl group is far superior to one [26]. Lastly, methoxylation of OH substituents can either attenuate or enhance activity [22]. 

The antibacterial concentrations of (iso)flavonoid derivatives have nevertheless been exhaustively covered in the literature and numerous SAR studies have identified important pharmacophore characters. However, mining these values from the literature to perform a QSAR study has not yet been attempted. The feasibility of this approach can be confirmed by statistical results performance validation, providing an indication of the integrity of the external test set (MIC data). 

Thus, our first objective was to isolate, elucidate and screen the (iso)flavonoid derivatives against a range of bacteria associated with skin and gastrointestinal complaints and add to the existing repository of knowledge on antibacterial concentrations (MIC). Secondly, to use a comprehensive dataset that specializes in *Erythrina* in a QSAR analysis, compiled from values acquired in the current study and mined from the literature. Lastly, to report on any expansion of knowledge related to the chemistry of the species.

## 2. Results and Discussion

### 2.1. Chemistry and MIC of South African Erythrina lysistemon

In South Africa, several species of *Erythrina* are found, but the most common are *E. caffra* Thunb., *E. latissima* E.Mey. and *E. lysistemon*. The brightly red flowered *E. lysistemon* resembles *E. caffra*, but the two can be easily distinguished due to differences in curvature of the flower petals and color, because flowers of *E. caffra* are orange and curve back to expose the stamens [27]. The popularity of *E. lysistemon* in traditional medicine in southern Africa is reflected in the numerous vernacular names that have been recorded for this species. The most commonly used names are *mokhupye* (Northern Sotho), *umsinsi* (Swazi, Zulu), *nsisimbana* (Tsonga), *mophêthê* (Tswana), *muvhale* (Venda) and *umsintsi* (Xhosa). The bark is invariably offered for sale at informal medicine (*muthi*) markets.

**Figure 1:**
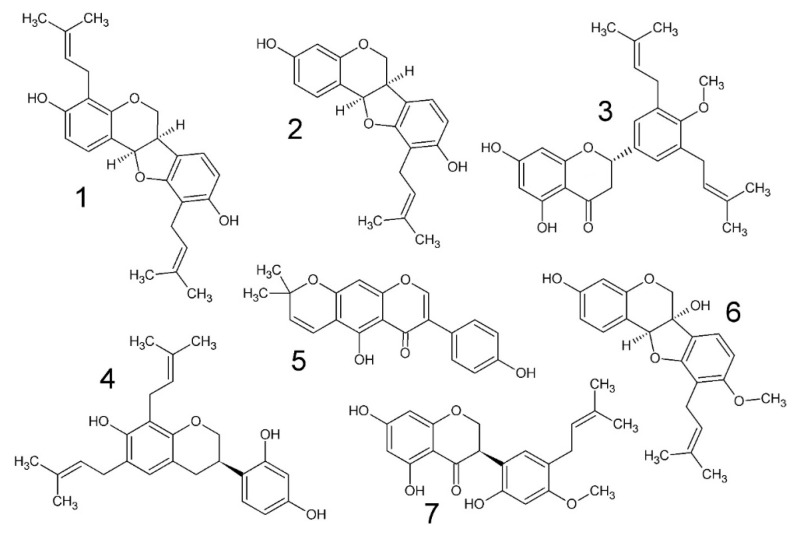
Chemical structures of compounds isolated in the current study from the stem bark of *Erythrina lysistemon*. erybraedin A (**1**), phaseollidin (**2**), abyssinone V-4’ methyl ether (**3**), eryzerin C (**4**), alpumisoflavone (**5**), cristacarpin (**6**) and lysisteisoflavone (**7**).

Extraction of the bark of *E. lysistemon* and flash chromatography lead to the isolation of one flavonoid (3), two (iso)flavonoids (5, 7), three pterocarpans (1, 2, 6) and one isoflavan (4). Three of the derivatives are di-prenylated (1, 3, 4), three are prenylated (2, 6, 7) and one is pyrano-cyclized over the phenolic ortho hydroxyl group (5), meaning the prenyl group is closed into a heterocycle which may attenuate the antimicrobial effects as indicated in previous studies [22]. The identity of the compounds are as follows; erybraedin A (1), phaseollidin (2), abyssinone V-4’ methyl ether (3), eryzerin C (4), alpumisoflavone (5), cristacarpin (6) and lysisteisoflavone (7). Compound 4 is reported from *E. lysistemon* for the first time. Compounds 2, 3, 5 and 6 have been previously studied for antimicrobial activity [28,29,30] against a narrower range of organisms using the accepted method by CLSI, which is the same method as that by Eloff [31]. Structures of 1–7 are illustrated in Figure 1. 

Compounds 1–7 are widely distributed across the genus. For example, cristacarpin (6) has also been isolated from *E. burana* Chiov. [15] and was demonstrated to be potentially active against cancer through their selective activity against DNA damage repair deficient yeasts. Compound 1 is also present in extracts from the Nigerian medicinal plant *E. mildbraedii* [3]. Compound 3 is also present in the Kenyan species *E. burttii* Baker.F. [14], which together with 5 demonstrated in vitro estrogenic [9] and anti-cholesterol gallstone formation activity [8]. 

The antimicrobial activities produced in the course of this study are presented in Table 1. MIC outcomes clearly demonstrate that this class of compound (prenylated (iso)flavonoid derivative) confers noteworthy antimicrobial activity against all bacteria, as defined by the criteria set out by Van Vuuren and Holl [32]. The activity of the crude extracts from both dichloromethane (DCM) and methanol (MeOH) may also be considered noteworthy against *S. aureus* and *S. epidermidis*, relative to values of crude extracts *per se*. While MIC values from isolated compounds demonstrated Gram-neutral inhibition, DCM and MeOH extracts discriminated against the Gram-positive species *S. aureus* and *S. epidermidis* with MIC values for the DCM extract at 104 and 4.8 μg/mL respectively, and values for the MeOH much higher at 125 μg/mL. Against *Bacillus cereus*, the MIC was only slightly higher (210–250 μg/mL). In contrast, values against the Gram-negative pathogens were in the range of 500–1000 μg/mL for extracts only, again with lower values from the DCM extract. The more potent activity of the DCM extract means that the active antimicrobial metabolites are of mid-range polarity and not likely to be glycosylated conjugates. This reinforces that 1–7 are the major contributors in antimicrobial outcomes.

It was expected that the hydrophilic barrier of Gram-negative cell walls would counteract the advantage of membrane penetrating ability of prenylated molecules isolated in the current study. But this was not the case. Indeed, the activity of this class of compound has been comprehensively studied against *E. coli* [22]. In that study, it was predicted that diprenylation would be associated with lower MIC values against *E. coli* but no experimental results were published at the time to confirm the author’s hypothesis. Nevertheless, the current study has corroborated this with MIC values of 2–5 μg/mL for diprenyl ‘ligands’ ortho positioned to a singular OH substituent, compared to values >20 μg/mL in monoprenylated molecules also with a single OH in the ortho position.

Erybraedin A (**1**) was identified in extracts of *E. latissima* and was one of the most active compounds tested in the study by Wanjala et al. [33], who used thin layer chromatography (TLC) bioautography to create MIC values. Abyssinone IV was the other most potent compound in that study, which is related to **3** of the current study, differing only by the presence of a methoxy group on the 4’carbon of **3**. The MIC value for **1** by Wanjana et al. [33] could not be compared to that of the current study, due to differences in methodology. However, the general trend is that **1**, **3** and **5** demonstrated more potent antimicrobial activity in other studies [28,33] as compared to our own, in contrast with **2**, which had a lower MIC value (10 μg/mL) against *S. aureus* as compared with the value published by Tanaka et al., [29], which was two-fold higher. In the study by Chukwujekwu et al. [28], 5 gave 3.9 μg/mL against both *S. aureus* and *E. coli*, but our study gave 31 and 125 μg/mL respectively. For the same two pathogens, **3** gave 31 and 3.9 μg/mL respectively [28], whereas 59 and 260 μg/mL was demonstrated in this study. 

Thus, due to small differences in experimentally determined MIC values across studies, a more robust assessment of SAR and QSAR will require a much bigger dataset than the seven isolated in the current study. Nevertheless, a priori assessment of the relative MIC values in Table 1 make sense from a SAR perspective. MIC values in Table 1 generally show that prenylation enhances activity and di-prenylation yet further. For example, **5**, which has no prenyl group, demonstrated generally lesser activity compared to the others (except **3** and **6**). It is hypothesized that prenylation enhances microbial membrane penetration by the attachment of a strongly lipophilic arm to the molecule [34]. From Table 1 and Figure 1, it appears that a hydroxyl (OH) group on the same aromatic ring as the prenyl moiety increases antimicrobial activity, but this activity is attenuated by methoxylation of that OH group. This is evident because the least activity is derived from **3** and **6**. The activity of **7** breaks this rule because another OH is present, in addition to a methoxy group, which in this case, does not attenuate activity. This observation is generally supported by other works on methoxylation in chalcones [19] and the studies by Rukachaisirikul et al. [4,5] which screened the same class of compounds against *Mycobacterium tuberculosis*. 

SAR and QSAR analysis of the entire dataset (74 compounds) against *S. aureus* corroborates this and elaborates on the importance of the position of ‘ligands’, the spatial distribution of ligands and the predicted vs. experimental outcome of methoxylation (Section 3.3 and Section 3.4). 

### 2.2. Brine Shrimp Lethality

Brine shrimp lethality testing following the method set out by Vanhaecke et al., [23] demonstrated that three of the compounds (**1**–**3**) and the crude extracts have low toxicity against brine shrimp and no toxicity at 1 mg/mL. For example, the MeOH and DCM extracts had 23.5% and 10.2% mortality at 24 h respectively and 37.1% and 16.7% at 48 h. Compounds **1**, **2** and **3** were as follows; 10.4%, 7.7% and 3.8% at 24 h and 19.1%, 22.9% and 14.0% at 48 h respectively. Unfortunately, **4**–**7** were exhausted in antimicrobial testing and were not screened for brine shrimp lethality in the current study. However, crude extracts demonstrated no toxicity, again with no lethality at 1 mg/mL, so it is feasible that screening of **1**–**3** is a reflection of the general lethality of the other metabolites. However, in a separate study, lethality (10 μg/mL) was measured for **5** [17], which is the most chemically unique compound isolated in this study, since the prenyl group is pyranocyclized. Thus, this compound needs to be examined further to confirm the published result. Furthermore, in a separate study, the compound phaseollidin (**2**) was demonstrated to be toxic against three cell lines [15]. 

### 2.3. Structure Activity Relationships

**Figure 2:**
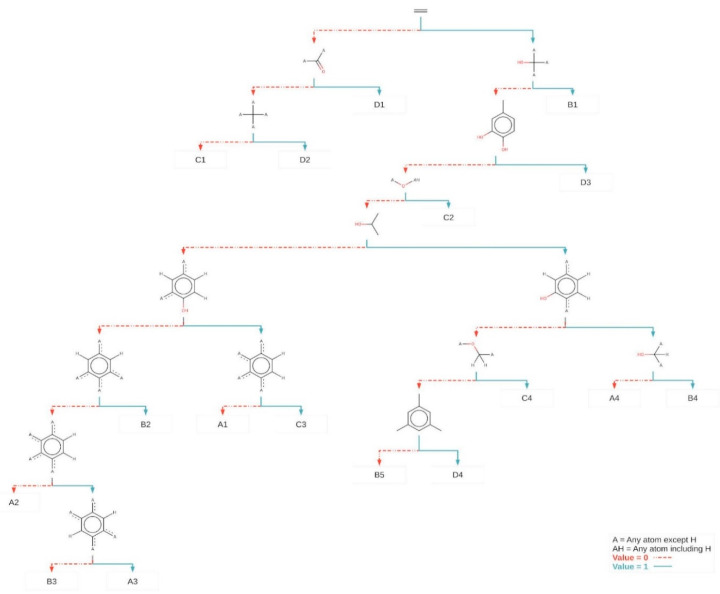
Discriminating Klekota-Roth fragments identified by the decision tree. The fragments were selected by the decision tree to discriminate the four classes of activity (A, B, C and D). The ‘A’ symbol in chemical structures indicates an atom that is not hydrogen (e.g., carbon or oxygen), and hydrogen atoms (whether shown or implied). The ‘AH’ symbol indicates an atom can be hydrogen, carbon or oxygen. Arrows indicate the presence of a particular fragment and dotted arrows indicate its absence. The nodes that contain the fragments are classified and sequential. The fraction of active compounds is listed on each end node (e.g., Class A, was found on four end nodes: A1, A2, A3 and A4 (Section 2.4)).

The 74 flavonoids were partitioned using a decision tree (Figure 2). In this tree, the ‘root’ node corresponds to the total set of flavonoids. The flavonoids were successively divided into smaller and smaller subsets, according to the presence or absence of a ‘distinctive’ fragment. The ‘X’ symbol indicates an atom that is not hydrogen and all hydrogen atoms in fragments containing ‘X’ (shown or implied) must be exactly the same. All other fragments have unspecified hydrogen and non-hydrogen atom substitution patterns. At each node in the tree, the J48 algorithm chooses the fragment that most effectively divides its set of samples into subsets of a class [35,36,37]. At the conclusion of the process, the selected fragments in the tree nodes may be the properties or characteristics of the class, such as, for example, the pharmacophore of chemical structures or the fraction that prevents biological activity. 

**Figure 3:**
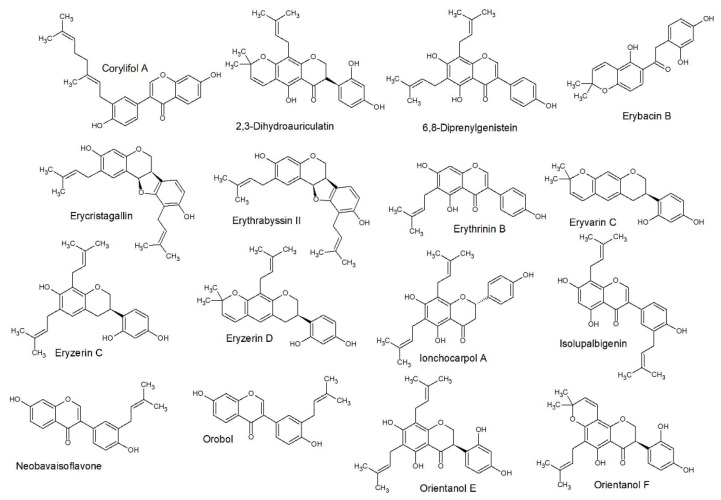
Structure activity relationship (SAR) predicted class A structures: This gave a similar class prediction as compared to QSAR predicted, which gave 77% accuracy (Figure 7).

**Figure 4:**
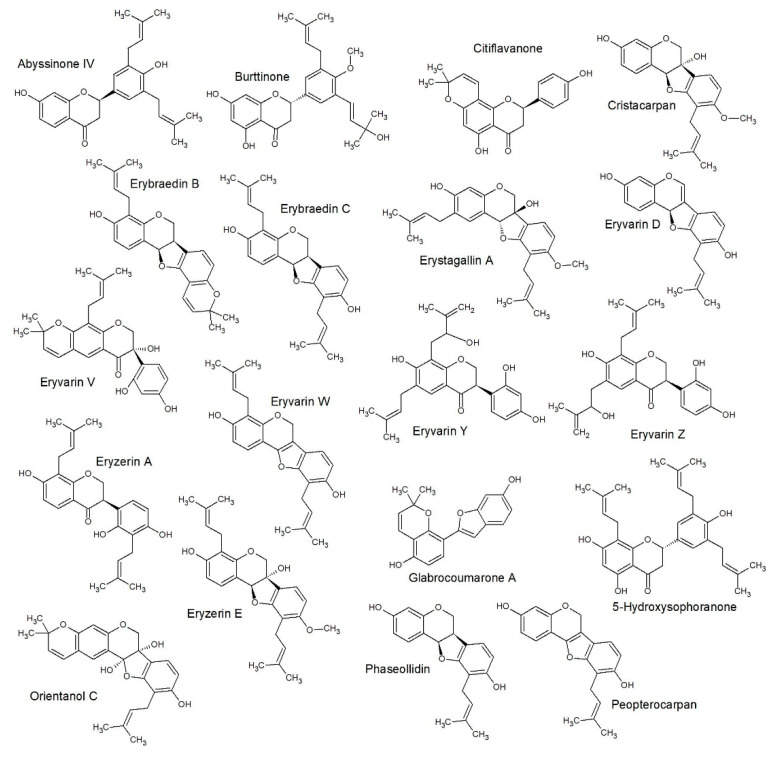
SAR predicted class B structures: This gave a similar class prediction as compared to QSAR predicted, which gave 77% accuracy (Figure 7).

**Figure 5:**
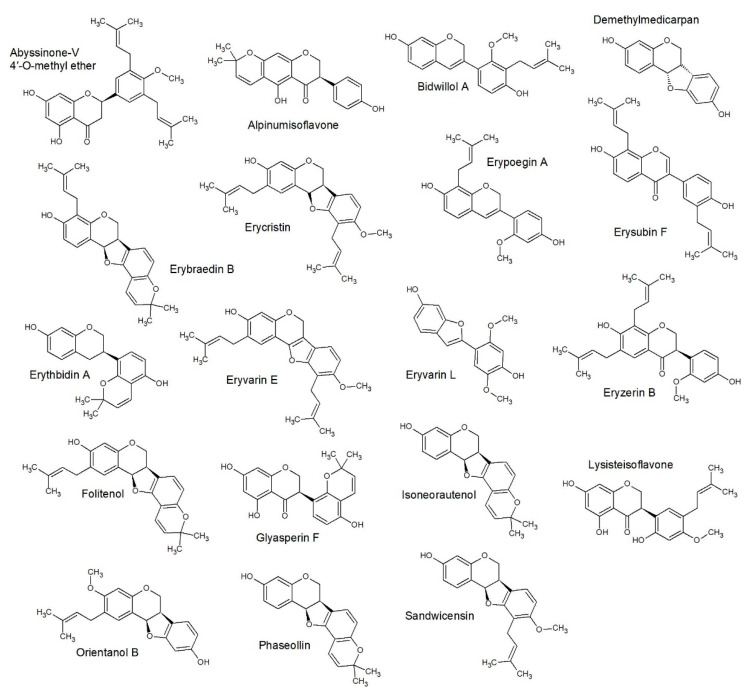
SAR predicted class C structures: This gave a similar class prediction as compared to QSAR predicted, which gave 77% accuracy (Figure 7).

**Figure 6:**
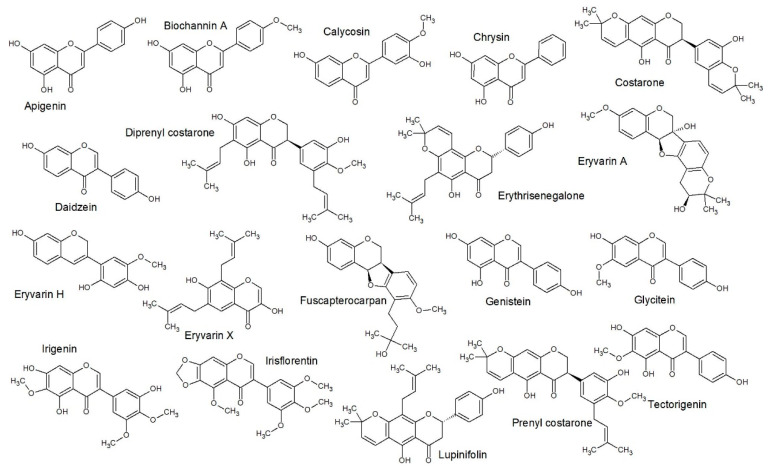
SAR predicted class D structures: This gave a similar class prediction as compared to QSAR predicted, which gave 77% accuracy (Figure 7).

The decision tree correctly classified 57 (accuracy of 77.03%) of the flavonoids and incorrectly classified 17 (error of 22.97%) with Cohen’s kappa of 0.69. The structures of each group, as classified by SAR only, are included as Figure 3 (class A), Figure 4 (class B), Figure 5 (class C) and Figure 6 (class D). 

At the first level of the decision tree (Figure 2), it was possible to separate some less active compounds (C1, D1 and D2) since the compounds classified as C and D have MIC equal to or greater than 25.0. Nevertheless, (iso)flavonoids and derivatives are differentiated based on their chemical configuration, giving a class system that reflects biosynthetic origins, but was initially inspired by antimicrobial activity. Since the class system relies heavily upon the position of the substituents on rings A, B and C, a strong correlation to activity should be evident [38]. For example, from the sixth level of the decision tree, the main distinct fragments are indicating the distribution of ligands (prenyl and OH groups) in ring A, after the previous nodes. 

Previous studies also verified the importance of the distribution and constitution of the ligands in this ring in the context of antimicrobial outcomes. It can be seen that activity is increased by the presence of prenyl groups at positions 6 or 8 in ring A and 3 ‘or 5’ in ring B [19,39]. The presence of hydroxyl groups in positions 5 and 7 in ring A and just one in the para substituted position in ring B is a factor that contributes to antibacterial activity. Polar groups bind strongly to the region of polar phospholipids on the bacterial membrane, leaving the hydrophobic moieties sandwiched into the interior of the bilayer, anchored by the polar head and interacting with the alkyl chains of the phospholipids. Such structural factors would explain the rupture of the membrane, resulting in the bacteriolytic action of these compounds [40].

However, the SAR study also highlighted some unique structural features (fragments) in the dataset that were not classed reflective of their MIC value. This highlights the conservative biosynthesis of most class A compounds in *Erythrina.* The best to exemplify this is diprenyl costarone [41], which had an MIC value in the class A range but was keyed out in the decision tree to a class D. This is due to the presence of the 3’-prenyl, 4’-methoxy, 5’-hydroxy configuration of ring B, also seen on prenyl costarone and lysisteisoflavone. Due to the poor representation of these structures in the published literature, it is difficult to predict the effects of this character; however studies on methoxylation in chalcones and isoflavones depict both attenuation and enhancement of antimicrobial activity [22]. In this case, the unique ring C-diol sequence is probably only slightly changed by methoxylation of just one of the hydroxyl groups. Thus, the QSAR analysis gave a better approximation of activity of diprenyl costarone (class B). 

### 2.4. Quantitative Structure Activity Relationships

In order to build a good model to explain and predict flavonoid antimicrobial properties, it was decided to use pace regression. Pace regression improves on classical ordinary least squares (OLS) regression as it assesses the effect of each variable individually and sequentially uses cluster analysis to improve the statistical basis to estimate its contribution to regression [42,43,44]. 

Thus, it was possible to build a model by reducing previously ranked descriptors using the wrapper subset evaluator and linear regression (OLS) as learning scheme for only five descriptors, maintaining the golden rule of parsimony [45].

**Figure 7:**
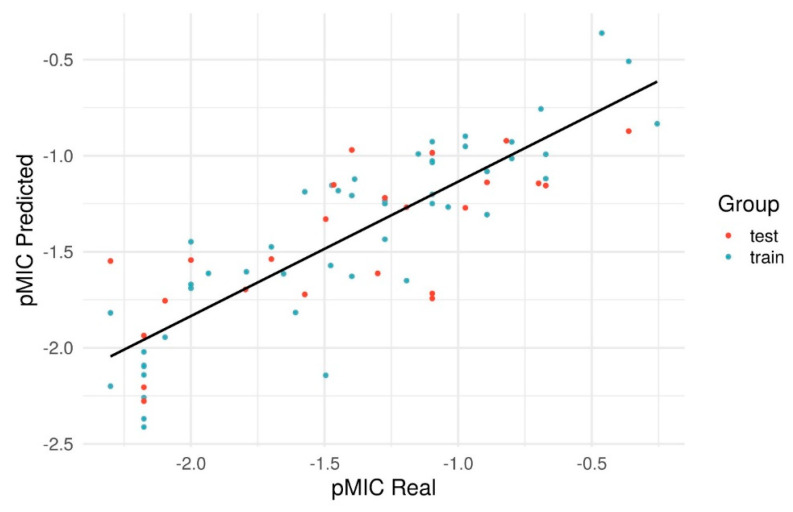
Plot of predicted versus experimental pMIC values for Pace Regression model.

In Figure 7, we show the linear relationship between the predicted-log of the MIC (pMIC) and the real pMIC and proven in Table 2. The numerical pMIC values can be seen in Table 3, Table 4, Table 5 and Table 6. The regression equation that obtained these results for the pMIC was:

pMIC = −1.812 − 0.6668*JGI6 + 2.0971*CrippenLogP − 0.6205*maxHBint6 + 1.5086*MDEO-11 − 1.5671*RDF70s, where JGI6 is the mean topological charge index of order 6. This descriptor represents a strictly topological quantity that plausibly correlates with the charge distribution within the molecule. In other words, the topological distance of the substituents plays an important role in determining the inhibitory activity. The negative coefficient of JGI6 indicates that the more substituents with a path length of 6, the lower the inhibitory activity [46].

CrippenLogP is the Crippen’s LogP. This descriptor is considered an informative parameter of the solubility tendency of a flavonoid. Once in the culture medium (e.g., aqueous solution), it is distributed between nonpolar structures (e.g., cell membranes) and aqueous solutions (intracellular fluids). The lower the CrippenLogP, the more hydrophilic the flavonoid is and the greater its tendency to dissolve in the aqueous phase. On the other hand, the higher the CrippenLogP, the greater the lipophilicity of the flavonoid. The positive CrippenLogP coefficient indicates that lipophilic flavonoids increase inhibitory activity [47].

maxHBint6 is the maximum e-state descriptors of strength for potential hydrogen bonds of path length 6. This descriptor is related to the electrotopological state of hydrogens capable of making a hydrogen bond with a path length of 6 and which may be involved in intermolecular contacts and interactions and, in addition, contribute to the general values of biological and physico-chemical properties. The negative coefficient of maxHBint6 indicates that the more strength for potential hydrogen bonds of path length 6, the lower the inhibitory activity [48].

MDEO-11 is molecular distance edge between all primary oxygens. The molecular distance limit between all primary oxygen has a good correlation with the physicochemical properties of the compounds. Molecular distance edge (MDE) vectors can be correlated with many physical properties and/or dynamic functions, such as Gibbs free energy, heat capacity, molar volume, molar refraction and so on. In particular, the presence of primary oxygen is related to intermolecular interactions of hydrogen bonding and the antioxidant and pro-oxidant capacity of the flavonoid. The positive coefficient of MDEO-11 indicates that the greater the total distance of all primary oxygen, the greater the inhibitory activity of the flavonoid [49,50].

RDF70s is radial distribution function-070/weighted by relative I-state. This is the only descriptor in the model that carries the three-dimensional information of the structure, the RDF descriptors in this case uses the property of the molecular intrinsic state (which takes into account the higher quantum level atoms and valence value) in a radius of 0.7 angstrom [51,52].

The statistical parameters found for the model were R² = 0.778, Q² = 0.727 and P² = 0.555 (Table 2). Values of R² (quality of fit) >0.6, Q² (robustness) and P² (predictive capacity) must be greater than 0.5 for a relevant prediction model [50].

The descriptors selected above were used for building the pace regression (PR). The PR was built on Weka 3.8.4, based on the selection of descriptors data and values of pMIC. The empirical Bayes estimator and standard parameters of software were used. The values of coefficient of determination (R^2^), mean absolute error (MAE) and root mean squared error (RMSD) were evaluated to measure the goodness of fit of the models; the values of coefficient of determination for cross-validation (Q^2^), MAE and RMSD were determined on leave-one-out-validation to evaluate the robustness, validity of the models and internal predictivity; and test the remainder to do external validation with data not used in the PR model development (P^2^, MAE and RMSD). To do an additional validation for the models, the scramble test was performed with the pMIC randomly changed on the dataset.

In order to detect overfitting, the use of scrambling is a useful diagnostic tool. The mixing is performed by randomly mixing the experimental activity of all compounds. The resulting data set should be evaluated as in the original training set. The proof that the predictive power of the model is achieved is provided when the scrambling analysis results in a coefficient of determination values below 0.5 [50]. This is expected for a good predictive model, because randomly mixing the experimental values of biological activity completely removes the relationship between structures and activity [50]. Thus, the scrambling test validated the PR model without overfitting. 

Extending the extrapolation is a simple approach to define the applicability of the domain. In the current study, we define the use of two types of algorithms, the first algorithm was based on the calculation of leverage and the second on the Euclidean distance. Both always compared the training set and the external test set [53,54,55]. The external test set proved to be reliable in both algorithms.

## 3. Materials and Methods

### 3.1. Materials

Stem bark of *E. lysistemon* was harvested from a specimen growing in the Johannesburg Faraday muthi market and a flowering voucher specimen of the tree (*Khumalo 1*) was lodged at the University of Johannesburg Herbarium (JRAU). The identity of the specimen was confirmed by muthi market traders and an experienced legume taxonomist (Ben Erik Van Wyk). The specimen was collected on 30 March 2016. 

### 3.2. Extraction

To obtain crude extracts for antimicrobial testing, dried and powdered bark (1 g) was soaked in either methanol or dichloromethane for 48 h at room temperature. The extracts were filtered through Whatman 1 filter paper and the solvent allowed to evaporate completely. Sample dry weights of 0.137 g (methanol) and 0.031 g (dichloromethane) were obtained, giving a yield of 13.7% and 3.1% respectively. 

### 3.3. Compound Isolation and Structural Elucidation

Dried and powdered bark (291.7 g) was extracted in dichloromethane for 48 hrs. The solvent was then filtered through celite and evaporated completely to afford 9.33 g of extract. Dry flash chromatography over silica gel (v/v of 20% acetone, 20% ethyl acetate, 60% pet ether) was used to clean the crude extract to 0.58 g, where the majority of the more polar compounds adhered to the silica gel. The 0.58 g sample was then loaded onto a silica gel column with starting mobile phase of 10% ethyl acetate in 90% toluene (v/v). This was incrementally increased to 30% ethyl acetate in 70% toluene (v/v). Thin layer chromatography on aluminum backed plates was used to guide fraction combining (20% ethyl acetate, 80% pet ether) and seven compounds were collected. The NMR spectra for the seven compounds were generated on a 500 Mhz Bruker Avance (Bruker, Germany) using standard Bruker pulse sequences, and the ^13^C and ^1^H spectra were matched to published values; 1 (erybraedin A, 93.7 mg) was matched to spectra reported by Mitscher et al. [3], 2 (phaseollidin, 13.4 mg) was matched to spectra by Dagne et al. [15], 3 (abyssinone V-4’ methyl ether, 23.6 mg) was matched to spectra by Yenesew et al. [14], 4 (eryzerin C, 32.6 mg) to spectra by Tanaka et al. [18], 5 (alpumisoflavone, 16.4 mg) to spectra by Juma and Majinda [17], 6 (cristacarpin, 11.6 mg) to spectra by Dagne et al. [15] and 7 (lysisteisoflavone, 17.6 mg) in El-Masry et al. [16].

### 3.4. Antimicrobial Studies

#### 3.4.1. Culture Preparation

The bacterial strains for antimicrobial screening were selected based on the gastrointestinal and topical traditional uses as recorded by published records on traditional use [6]. These include Gram-negative strains; *Escherichia coli* ATCC 8739, *Pseudomonas aeruginosa* ATCC 27853, and Gram-positive strains; *Staphylococcus aureus* ATCC 25923, *Staphylococcus epidermidis* ATCC 12228 and *Bacillus cereus* ATCC 11175. 

#### 3.4.2. Determination of Minimum Inhibitory Concentrations (MIC)

The minimum inhibitory concentration (MIC) method [31] was used to determine the susceptibility of test pathogens to crude extracts and isolated compounds. A starting concentration of 8 mg/mL was used for crude extracts, while the starting concentration for isolated compounds was 1.25 mg/mL. Acetone was used for dissolving compounds and extracts. Using aseptic technique, 100 μL of Tryptone soya broth was introduced in each well of a 96 well micro-titer plate. Crude extracts as well as isolated compounds (100 μL) were transferred to the first row of the micro-titer plate together with acetone. Acetone was also included separately as a negative control (8 mg/mL) and ciprofloxacin (0.01 mg/mL) as the positive control. Serial dilutions were performed, followed by the addition of 100 μL of the culture (at 1 × 10^6^ CFU/mL) to all the wells. Each plate was then sealed with a sterile adhesive sealing film. The plates were incubated overnight at 37 ℃, then 40 μL of *p*-iodonitrotetrazolium chloride (INT) was added as a growth indicator to each well of the micro-titer plates. The MIC values were recorded as the lowest concentration of the tested compounds and crude extracts by the appearance of the typical red color indicating visible microbial growth. The MICs were completed in duplicate and repeated three times to confirm accuracy of results and values are presented as an average. Accuracy of results were confirmed if MIC values were within one order of magnitude (one dilution), which gives a standard deviation ranging from 22–37% of the MIC value. 

### 3.5. SAR and QSAR Analysis

A summary of 74 MIC values against *S. aureus* for isoflavone and flavone derivatives was compiled. Seven of these values were determined in the current study and the other 67 were mined from the literature. Positive selection criteria for publication data included: (a) the study was focused on a species of *Erythrina*, (b) MIC data must have been determined using the currently accepted method by the clinical laboratory standards institute [20] and (c) data must be judged as authentic. Studies were excluded if they only used disc diffusion. On a few occasions, studies were not included due to unrealistically low MIC values. Data included in the current study were taken as either true or averaged from a set of values out of the following studies: [3,4,18,24,28,29,30,41,56,57,58,59,60,61,62]. For convenience, the new isoflavanones from *Erythrina costaricensis* Micheli, in the study by Tanaka et al. [41], were named (1) diprenyl costarone, (2) prenyl costarone and (3) costarone, in the same numerical order as used in that study. 

#### 3.5.1. Structure Optimization

The chemical structures were drawn, normalized, standardized and the 3D coordinates were calculated by Marvin 19.27 [63]. Energy minimization of chemical structures was performed using the semi-empirical PM7 method within software MOPAC2016 [64] (Parameters: PM7 MMOK XYZ BONDS GNORM = 0.0001 GRAPHF PDBOUT). To change the file formats (mol to mop and pdb to sdf), the software Open Babel 2.4.0 [65,66] was used.

#### 3.5.2. Descriptors Calculation

In order to perform the SAR studies, the Klekota-Roth fragments [67] were calculated (*n* = 4860) and for the QSAR studies all types of molecular descriptors (*n* = 1874), e.g., constitutional, topological, geometrical, electronic and, physicochemical descriptors were calculated with the aid of software Padel 2.21 [68].

#### 3.5.3. SAR Studies

In order to know which side chains could indicate potential activity or improve activity, a SAR (structure–activity relationship) study was performed. Thus, the activities were classified into four classes (A—1.8 to 9.4, B—10.9 to 24.4, C—25.0 to 86.0 and D—100 to 200). The raw MIC data according to class are given as Table 3 (class A), Table 4 (class B), Table 5 (class C) and Table 6 (class D). All Klekota-Roth fragments which had zero variance (fragments that could not differentiate between flavonoids) were excluded and a decision tree using the J48 algorithm was built with the software Weka 3.8.4 [43,44].

#### 3.5.4. QSAR Studies

A previously mentioned, the MIC values for each flavonoid derivative were based on previous in vitro studies and those demonstrated by the current study. Values were normalized to pMIC values (−logMIC) in order to facilitate comparisons. To develop an activity prediction model and a QSAR study, the structures were divided into two groups (training set *n* = 50 and test set *n* = 24). The descriptors data were normalized with the software Weka 3.8.4 using the scale 1 to 0. After the normalization, the important descriptors for the values of pMIC were ranked using wrapper subset evaluator and linear regression as the learning scheme. In order to obtain a parsimonious model, five descriptors were selected based on the physicochemical information and Pearson correlation between them not exceeding 0.65. The model applicability domain must be defined to flag compounds in the test set for which predictions can be reliable or unreliable. Thus, two analyzes were performed: the applicability domain (APD) based on the Leverages and APD based on the Euclidean distances [53,54,55].

## 4. Conclusions

The results of the current study demonstrate that the prenylated (iso)flavonoid derivatives are important metabolites in achieving the antimicrobial outcomes from extracts of *Erythrina*, particularly in traditional use systems. However, these findings are unsurprising, considering the evidence that has been accumulated via studies during the last few decades. It is nevertheless surprising that these well-known antimicrobial compounds have not been of greater use in industry. The literature dealing with toxicity has been overwhelmingly neglected and a clearer understanding needs to be established in the promotion of their use. 

Of greater interest is in being able to quantitatively predict the potency of structures against specific pathogenic bacteria. To date, only one other study has done this, which focused on *E. coli* and *L. monocytogenes* using a lower number of samples (30). Due to the low degree of specificity of action in prenylated (iso)flavonoids and pterocarpans, there is not much difference between the susceptibility of bacterial species and structural requirements for potency in natural products. Since strain types were not considered a discriminating factor in MIC value mining, many of the values included in the current study were derived from the resistant strains (MRSA) and the values were combined with non-resistant strains. Validation of the dataset demonstrated that variation across strains was smaller than experimental error. From this, it is clear that resistance mechanisms in *S. aureus* are not effective against the less specific mechanisms of inhibition by natural products, such as the prenylated isoflavones and derivatives. 

There were some limitations in the current study. By using a higher number of replicates (74) and including a greater diversity of structural characters, it was realized that the effects of methoxylation of single dihydroxy prenylated aromatic rings are not yet predictable. This opens a potential research niche for exploration of primary and secondary oxygens on aromatic rings in prenylated (iso)flavonoids, (iso)flavans, pterocarpans, stilbenes or chalones. 

## Figures and Tables

**Table 1:** Mean minimum inhibitory concentration (MIC) values (μg/mL) of crude extracts and isolated compounds (**1–7**) from stem bark of *Erythrina lysistemon*. The pure compounds tested are erybraedin A (**1**), phaseollidin (**2**), abyssinone V-4’ methyl ether (**3**), eryzerin C (**4**), alpumisoflavone (**5**), cristacarpin (**6**) and lysisteisoflavone (**7**). + Cont = positive control = Ciprofloxacin. Standard deviation values ranged from 22%–37% of the MIC value.

Pathogens	Extracts		Pure Compounds							
	DCM	MeOH	1	2	3	4	5	6	7	+Cont
*Bacillus cereus* ATCC 11175	250	210	1	10	26	10	31	156	2	0.020
*Staphylococcus aureus* ATCC 25923	104	125	2	10	59	5	31	156	62	0.078
*S. epidermidis* ATCC 12228	5	125	2	5	117	2	125	412	26	0.078
*Escherichia coli* ATCC 8739	667	1000	2	20	260	5	125	625	6	0.078
*Pseudomonas aeruginosa* ATCC 27853	500	830	20	20	260	5	20	78	31	0.078

**Table 2:** Statistical results of performance validation.

Step	Coefficient of Determination	MAE	RMSD	R² Scramble	MAE Scramble	RMSD Scramble
Train	0.778	0.212	0.261	0.037	0.451	0.544
LOO-CV	0.727	0.238	0.290	−0.154	0.500	0.595
Test	0.555	0.298	0.359	−0.045	0.460	0.551

**Table 3:** Flavonoid and (iso)flavonoid derivatives categorized as class A according to experimental MIC (0–10 μg·mL^−^). Class predicted by SAR is created from structural fragments, whereas QSAR predicted MIC uses experimental MIC in the algorithm.

Compounds	Class	Class Predicted SAR	pMIC	pMIC Predicted	Experimental MIC	QSAR Predicted MIC	Class Predicted QSAR
Isolupalbigenin	A	A1	−0.36	-0.87	2.3	7.4	A
Corylifol A	A	A1	−0.7	−1.14	5.0	13.8	B
Eryzerin C	A	A2	−0.67	−1.16	4.7	14.5	B
lonchocarpol A	A	A2	−0.8	−0.93	6.3	8.5	A
Orientenol E	A	A2	−0.82	−0.92	6.6	8.3	A
Diprenylgenistein	A	A2	−0.89	−1.08	7.8	12.0	B
Erythrinin B	A	A2	−0.89	−1.31	7.8	20.4	B
Erythrabyssin−II	A	A3	−0.26	−0.83	1.8	6.8	A
Erycristagallin	A	A3	−0.46	−0.36	2.9	2.3	A
Orientanol F	A	A4	−0.89	−1.14	7.8	13.8	B
Erybacin B	A	A4	−0.97	−0.9	9.3	7.9	A
Eryzerin D	A	A4	−0.97	−1.27	9.3	18.6	B
Eryvarin W	A	B3	−0.36	−0.51	2.3	3.2	A
Erybraedin A	A	B3	−0.69	−0.76	4.9	5.8	A
Orientanol B	A	C2	−0.67	−0.99	4.7	9.8	A
Erycristin	A	C2	−0.8	−1.01	6.3	10.2	A
Bidwillol A	A	C2	−0.97	−0.95	9.3	8.9	A
Diprenyl costarone	A	D3	−0.67	−1.12	4.7	13.2	B

**Table 4:** Flavonoid and (iso)flavonoid derivatives categorized as class B according to experimental MIC (10−20 μg·mL^−^). Class predicted by SAR is created from structural fragments, whereas QSAR predicted MIC uses experimental MIC in the algorithm.

Compounds	Class	Class Predicted SAR	pMIC	pMIC Predicted	Experimental MIC	QSAR Predicted MIC	Class Predicted QSAR
Neobavaisoflavone	B	A1	−1.3	−1.61	20.0	40.7	C
Dihydroauriculatin	B	A4	−1.15	−0.99	14.1	9.8	A
Eryvarin C	B	A4	−1.27	−1.44	18.6	27.5	C
Erystagallin A	B	B1	−1.04	−1.27	11.0	18.6	B
Eryzerin E	B	B1	−1.19	−1.27	15.5	18.6	B
Orientanol C	B	B1	−1.19	−1.65	15.5	44.7	C
Eryvarin V	B	B1	−1.27	−1.22	18.6	16.6	B
5−Hydroxysophoranone	B	B2	−1.1	−0.93	12.6	8.5	A
Abyssinone IV	B	B2	−1.1	−1.25	12.6	17.8	B
Erybraedin C	B	B3	−1.1	−1.03	12.6	10.7	A
Eryvarin D	B	B3	−1.1	−0.98	12.6	9.5	A
Ptorepterocarpan	B	B3	−1.1	−0.99	12.6	9.8	A
Eryzerin A	B	B3	−1.27	−1.25	18.6	17.8	B
Phaseollidin	B	B3	−1.39	−1.12	24.5	13.2	B
Eryvarin Y	B	B4	−1.27	−1.23	18.6	17.0	B
Citflavanone	B	B5	−1.1	−1.74	12.6	55.0	C
Glabrocoumarone A	B	B5	−1.1	−1.2	12.6	15.8	B
Erybraedin B	B	C4	−1.1	−1.04	12.6	11.0	B
Lupinifolin	B	D4	−1.1	−1.72	12.6	52.5	C

**Table 5:** Flavonoid and (iso)flavonoid derivatives categorized as class C according to experimental MIC (20−100 μg·mL^−^). Class predicted by SAR is created from structural fragments, whereas QSAR predicted MIC uses experimental MIC in the algorithm.

Compounds	Class	Class Predicted SAR	pMIC	pMIC Predicted	Experimental MIC	QSAR Predicted MIC	Class Predicted QSAR
Orobol	C	A1	−1.93	−1.61	85.1	40.7	C
Eryvarin Z	C	B4	−1.57	−1.19	37.2	15.5	B
Eryravin L	C	C1	−1.4	−1.63	25.1	42.7	C
Demethylmedicarpin	C	C1	−1.7	−1.54	50.1	34.7	C
Erypoegin A	C	C2	−1.4	−1.21	25.1	16.2	B
Eryvarin E	C	C2	−1.4	−0.97	25.1	9.3	A
Sandwicensin	C	C2	−1.47	−1.15	29.5	14.1	B
Abyssinone−V 4−O−methyl ether	C	C2	−1.65	−1.61	44.7	40.7	C
Lysisteisoflavone	C	C2	−1.79	−1.6	61.7	39.8	C
Eryzerin B	C	C2	−1.8	−1.7	63.1	50.1	C
Glabrol	C	C3	−1.47	−1.15	29.5	14.1	B
Erysubin F	C	C3	−1.7	−1.47	50.1	29.5	C
Folitenol	C	C4	−1.45	−1.18	28.2	15.1	B
Alpinumisoflavone	C	C4	−1.48	−1.57	30.2	37.2	C
Erythbidin A	C	C4	−1.5	−1.33	31.6	21.4	B
Glyasperin F	C	C4	−1.5	−2.14	31.6	138.0	D
Phaseolin	C	C4	−1.57	−1.72	37.2	52.5	C
Prenyl costarone	C	D3	−1.61	−1.82	40.7	66.1	C

**Table 6:** Flavonoid and (iso)flavonoid derivatives categorized as class D according to experimental MIC (100–200 μg·mL^−^). Class predicted by SAR is created from structural fragments, whereas QSAR predicted MIC uses experimental MIC in the algorithm.

Compounds	Class	Class Predicted SAR	pMIC	pMIC Predicted	Experimental MIC	QSAR Predicted MIC	Class Predicted QSAR
Burttinone	D	B1	−2.1	−1.76	125.9	57.5	C
Cristacarpin	D	B1	−2.1	−1.94	125.9	87.1	D
Isoneorautenol	D	C4	−2	−1.45	100.0	28.2	C
Apigenin	D	D1	−2.18	−2.1	151.4	125.9	D
Biochanin A	D	D1	−2.18	−2.21	151.4	162.2	D
Calycosin	D	D1	−2.18	−2.41	151.4	257.0	D
Chrysin	D	D1	−2.18	−2.14	151.4	138.0	D
Daidzein	D	D1	−2.18	−2.28	151.4	190.5	D
Genistein	D	D1	−2.18	−2.02	151.4	104.7	D
Glycitein	D	D1	−2.18	−2.37	151.4	234.4	D
Irigenin	D	D1	−2.18	−1.94	151.4	87.1	D
Irisflorentin	D	D1	−2.18	−2.26	151.4	182.0	D
Tectorigenin	D	D1	−2.18	−2.09	151.4	123.0	D
Eryvarin A	D	D2	−2.3	−2.2	199.5	158.5	D
Fuscapterocarpan	D	D2	−2.3	−1.82	199.5	66.1	C
Costarone	D	D3	−2	−1.69	100.0	49.0	C
Eryvarin H	D	D3	−2	−1.54	100.0	34.7	C
Eryvarin X	D	D4	−2	−1.67	100.0	46.8	C
Erythrisenegalone	D	D4	−2.3	−1.55	199.5	35.5	C
